# Colon cancer combined with obesity indicates improved survival- research on relevant mechanism

**DOI:** 10.18632/aging.103972

**Published:** 2020-11-10

**Authors:** Zhou Yang, Xiyi Wei, Yitong Pan, Zhijun Min, Jingyuan Xu, Bo Yu

**Affiliations:** 1 Department of General Surgery, Shanghai Pudong Hospital, Fudan University Pudong Medical Center, Shanghai 201399, China; 2 Department of Urology, The First Affiliated Hospital of Nanjing Medical University, Nanjing 210009, Jiangsu, China; 3 Department of Bioinformatics, School of Biomedical Engineering and Informatics, Nanjing Medical University, Nanjing 211116, China; 4 Department of Gastroenterology, The Second Affiliated Hospital of Xi'an Jiaotong University, Xi'an 710004, China; 5 Department of General Surgery, Huashan Hospital Affiliated to Fudan University, Shanghai 201399, China

**Keywords:** obesity, colon cancer, hypoxia, immune checkpoints, TP53

## Abstract

Obesity contributes to the incidence of various tumors, including colon cancer. However, the impact of obesity on patients’ survival and related mechanisms remains unclear. Multi-omics data of 227 cases of colon cancer patients combined with clinical characteristics data were acquired from The Cancer Genome Atlas (TCGA) database. We confirmed obesity as an independent prognostic factor for improved overall survival of colon cancer patients. We demonstrated that hypoxia pathways were repressed in obese patients by regulating miR-210. Immune checkpoints PD-1 and LAG3 were also downregulated in obese patients, which indicated enhanced immune surveillance. The frequency of PIK3CA and KRAS mutations was decreased in obese patients. The sites and types of TP53 mutation were alternated in obesity patients. In conclusion, our research demonstrated the potential mechanisms of prolonged survival in colon cancer patients combined with obesity, which may provide potential value for improving the prognosis of colon cancer.

## INTRODUCTION

Colon cancer, with a mortality rate of 33%, is the third leading cause of cancer death; each year, nearly one million people suffer from this disease. Although early diagnosis might improve the prognosis, the overall survival rate of colon cancer is still poor. Nearly one million people suffer from it every year with a mortality rate of 33% [1]. The prognosis of colon cancer patients isnot satisfactory and survival is poor [2]. Therefore, it is urgent to explore more effective ways to prolong the survival of colon cancer patients.

Increasing evidence has indicated the association between obesity and increased risk of colon cancerr [3, 4]. However, previous research also suggested a decreased mortality rate of colon cancer in people of overweight and obesity. Similar affair also appears in other cancer and this phenomenon was termed “obesity paradox”. [5–7]. Not surprisingly, this paradox also exists in colon cancer. Decreased mortality risk was demonstrated in obese or overweight colon cancer patients [8–11]. However, the mechanism behind the paradox remains unclear. To verify and explore the mechanism behind this paradox in colon cancer, our research analyzed the multi-omics data from The Cancer Genome Atlas (TCGA) database.

## RESULTS

### Obesity is an independent prognostic factor for improved overall survival

**Figure 1:**
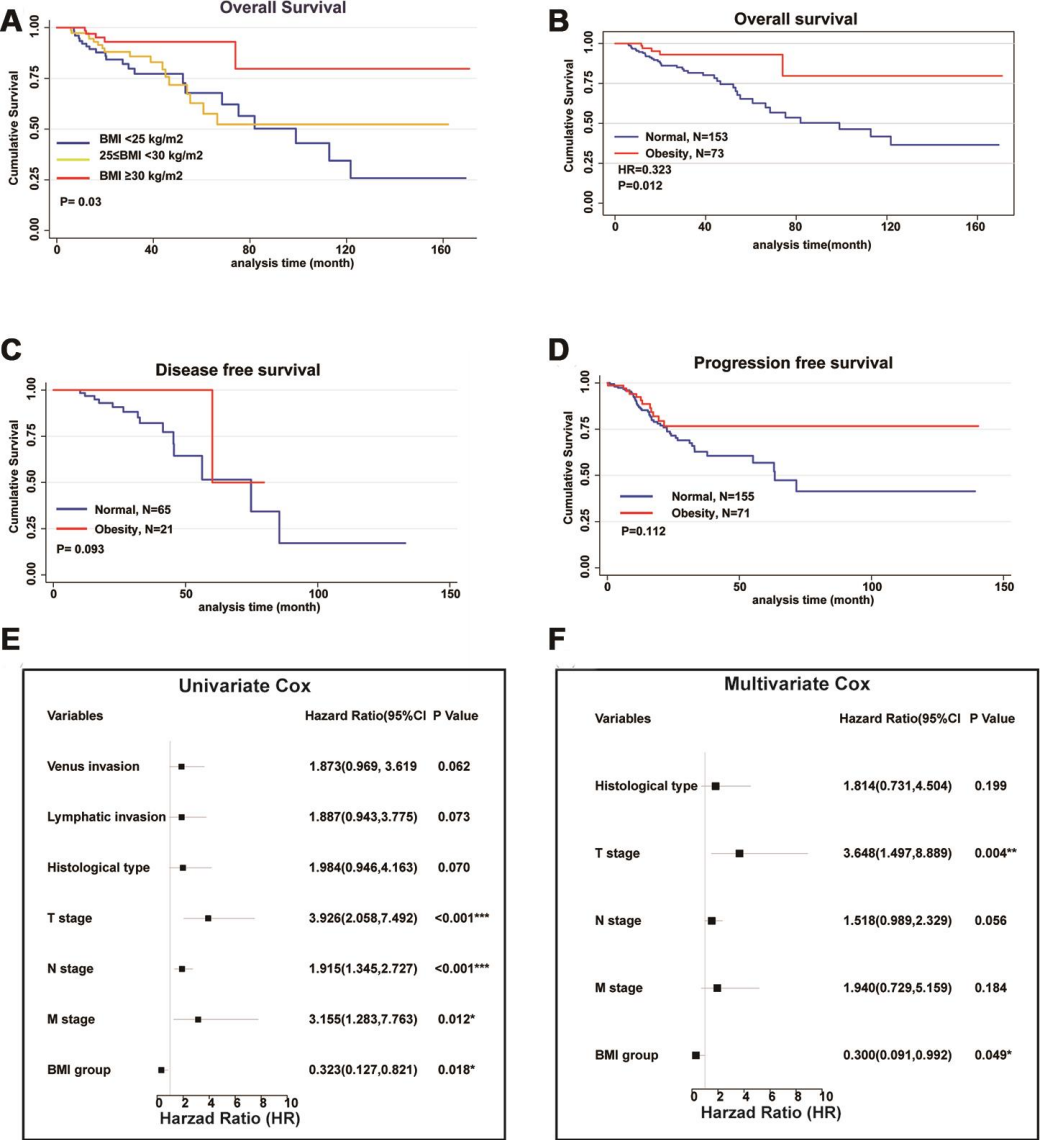
**Obesity is an independent prognostic factor for improved overall survival.** (**A**) The association of BMI and Overall Survival (OS) performed by Kaplan-Meier analysis. Patients were divided into 3 groups according to WHO standard (normal weight: BMI<25, overweight: 25≤BMI<30 and obesity: BMI≥30 kg/m^2^). (**B**) All patients redistributed into 2 groups (combined normal weight and overweight patients as normal group: BMI<30 *vs* obesity group: BMI≥30 kg/m^2^) and performed by Kaplan-Meier analysis. (**C**) Disease-Free Survival of normal group and obesity group performed by Kaplan-Meier analysis. (**D**) Progression-Free Survival of normal group and obesity group performed by Kaplan-Meier analysis. (**E**) Risk factors of OS analyzed by univariate Cox regression model. (**F**) Risk factors of OS analyzed by multivariate Cox regression model. (^*^p<0.05, ^**^p<0.01, ^***^p<0.001).

To investigate the relationship between BMI and Overall Survival (OS), we divided colon cancer patients in TCGA database into 3 groups according to the WHO standards (normal weight: 18.5≤BMI<25, overweight: 25≤BMI<30 and obesity: BMI≥30 kg/m^2^. There are only 2 cases of underweight patients, BMI<18.5 kg/m^2^, so they were eliminated) and performed with Kaplan-Meier analysis [12]. Both Compared to normal weight and overweight patients, obesity patients (BMI >=30 kg/m^2^) showed improved OS. However, no significant difference in OS was demonstrated between normal weight and overweight patients (Figure 1A). Subsequently, we combined normal weight and overweight patients as normal group and redistributed all patients into two groups (normal group: 18.5≤BMI<30kg/m^2^ *vs* obesity group: BMI≥30kg/m^2^), and performed Kaplan-Meier analysis repeatedly. The obesity group showed significantly better OS compared with normal group (*P* value=0.012, Figure 1B). Additionally, we also investigated the correlations of obesity with Disease-Free Survival (DFS) and Progression-Free Survival (PFS) respectively (Figure 1C, 1D). Though improved RFS and PFS were demonstrated in obesity group, p values were more than 0.05 (DFS: 0.093, PFS: 0.012). This might be due to the limited sample size. Furthermore, we investigated risk factors of OS via univariate and multivariate Cox regression model respectively. In univariate Cox regression model, Primary tumor (T), Regional lymph node (N), Distant metastasis (M) as well as BMI group (obesity group *vs* normal group, Hazard Ratio, HR=0.323, *P* value=0.018) were associated with OS (Figure 1E). In multivariate Cox regression model, only Primary tumor (T) and BMI group (HR=0.3, *P* value=0.049) were independent prognostic factors (Figure 1F). We also investigated the association of obesity and other characteristics of colon cancer patients, including age, gender, pathological stage, microsatellite status, and so on. Whereas BMI showed no association with all other characteristics (Table 1). In summary, we demonstrated obesity group ((BMI >=30 kg/m^2^) had improved OS compared with normal group (BMI<30kg/m^2^).

**Table 1:** The association of obesity and clinical characteristics.

	**Normal group (n=77) Number (percentage) or Medium±Interquartile** **Range**	**Obesity group (n=149) Number (percentage) or Medium±Interquartile Range**	**Pearson Chi-Square value/Z value**	**P value**
Sex (male)	86 (56.2%)	34 (46.6%)	1.842	.175
Lymphatic invasion	36 (26.1%)	26 (37.1%)	2.713	.100
Venus invasion	30 (22.1%)	16 (23.2%)	.034	.855
Histological type adenocarcinoma	126 (84.0%)	65 (89.0%)	1.015	.314
mucinous adenocarcinoma	24 (16.0%)	8 (11.0%)		
Polyps history	29 (25.0%)	14 (21.2%)	.334	.563
Pathological T stage			2.602	.457
T1	3 (2.0%)	0 (0.0%)		
T2	19 (12.4%)	13 (18.1%)		
T3	112 (73.2%)	50 (69.4%)		
T4	19 (12.4%)	9 (12.5%)		
Pathological N stage			1.382	.501
N0	90 (58.8%)	39 (53.4%)		
N1	35 (22.9%)	22 (30.1%)		
N2	28 (18.3%)	12 (16.4%)		
Pathological M stage			.122	.727
M0	111 (91.7%)	55 (93.2%)		
M1	10 (8.3%)	4 (6.8%)		
Microsatellites			4.481	.106
mss	94 (61.4%)	54 (75.0%)		
msi-l	29 (19.0%)	7 (9.7%)		
msi-h	30 (19.6%)	11 (15.3%)		
CEA level (ng/ml)	3±7.2	3±3.45	-1.091	.275

### Differentially expressed genes in obesity patients were closely related to tumor regulation

**Figure 2:**
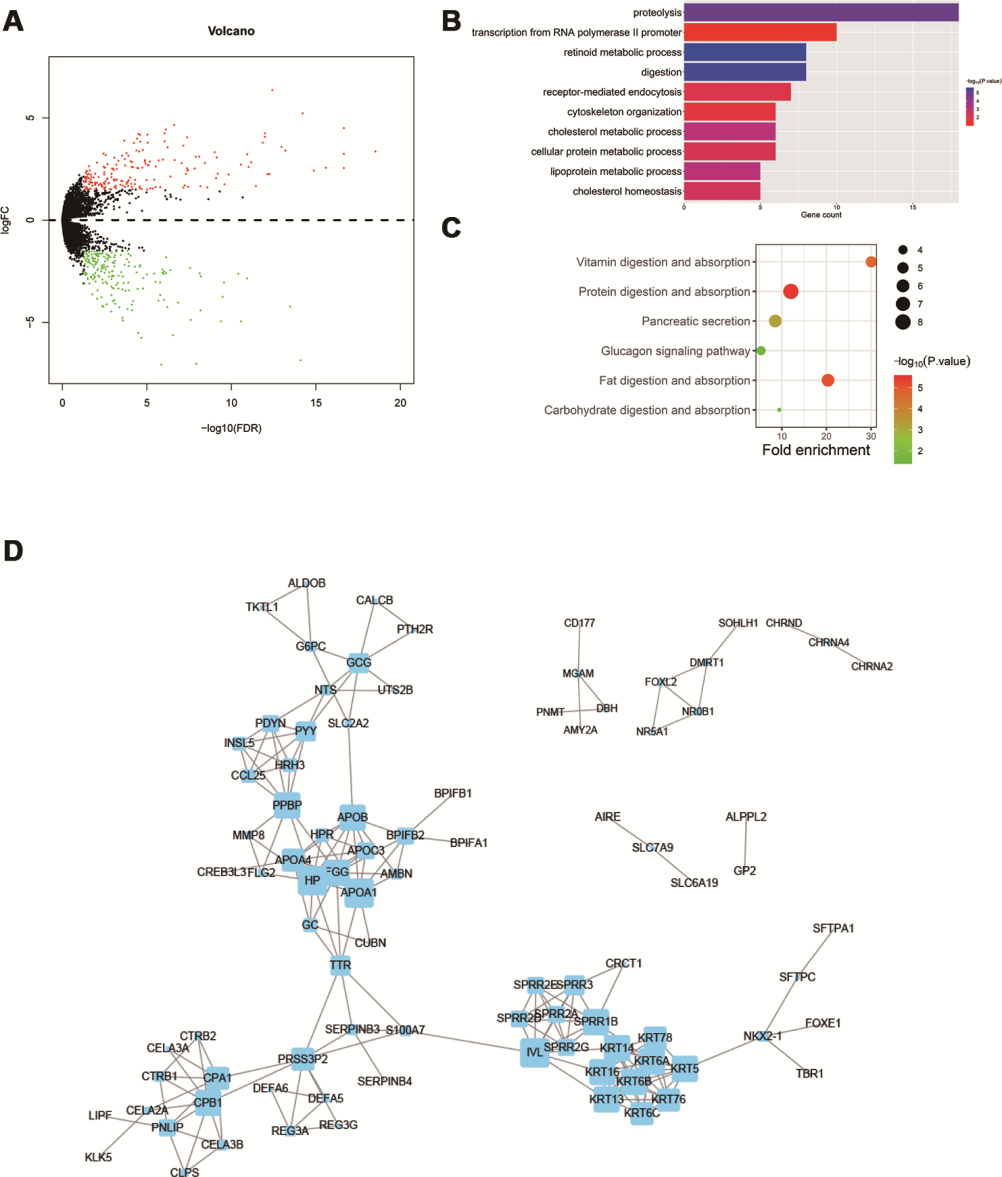
**Differentially expressed genes in obesity patients are closely related to tumor regulation.** (**A**) Differentially expressed genes (DEGs) between obesity and normal groups. (**B**) Gene Ontology (GO) analysis of DEGs. (**C**) KEGG pathway analysis of DEGs. (**D**) Protein-protein interaction (PPI) analysis of DEGs.

R software (“Limma” Package) was used to screen differentially expressed genes (DEGs) between normal and obesity groups based on transcriptome data. A total of 225 DEGs were confirmed and further performed with GO, KEGG, and PPI analysis respectively (Figure 2A). In both GO and KEGG analysis, nutrient digestion annotations and pathways were the most enriched, including vitamins, proteins, and so on (Figure 2B, 2C). In PPI analysis, we found 4 main modules, including small proline-rich protein 2 family (SPRR2s), Carboxypeptidase associated genes (CPA1, CPB1), Keratin family (KRTs) and Apolipoprotein family (APOA1, APOB, APOC3) (Figure 2D). All these families were reported to regulate the development of tumor.

### Obesity was associated with repressed hypoxia in colon cancer via inhibiting miR-210

**Figure 3:**
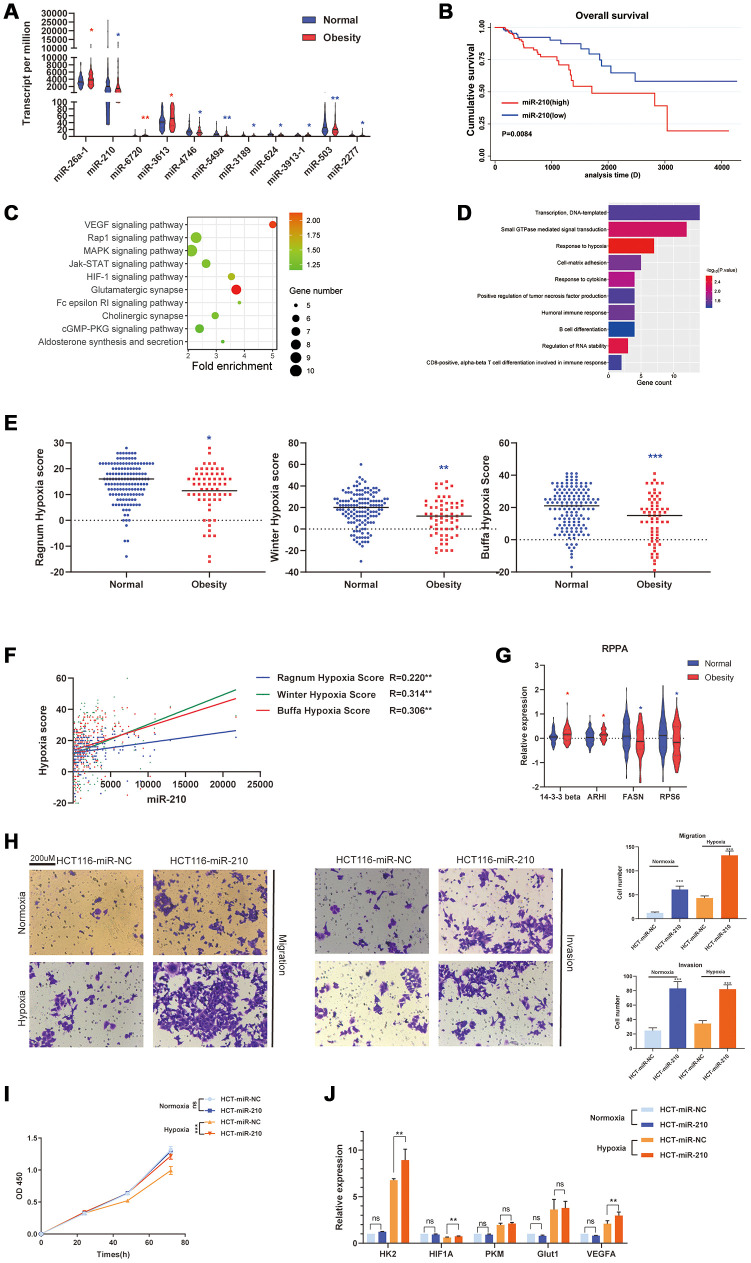
**Obesity was associated with repressed hypoxia in colon cancer via inhibiting miR-210.** (**A**) Differentially expressed miRNAs (DEMs) between obesity and normal groups. (**B**) The relationship of OS and miR-210 (divided by median of expression) performed by Kaplan-Meier analysis. (**C**) KEGG pathway analysis of miR-210-targeted genes (predicted by TargetScan database). (**D**) GO analysis of miR-210-targeted genes. (**E**) Hypoxia score of obesity and normal groups, derived from three different hypoxia scoring systems (Ragnum, Winter, Buffa hypoxia system). (**F**) The correlation of miR-210 and 3 different hypoxia scores analyzed by Pearson Correlation Coefficient. (**G**) Differentially expressed proteins between obesity and normal groups derived from Reverse Phase Protein Arrays (RPPA) data. (**H**) The invasion and migration ability of HCT-116 cells under hypoxia and normoxia. (**I**) The proliferation ability of HCT-116 cells under hypoxia and normoxia. (**J**) The expression of hypoxia associated genes of HCT-116 cells under hypoxia and normoxia. (^*^p<0.05, ^**^p<0.01, ^***^p<0.001).

Similarly, we also screened differentially expressed miRNA (DEMs) between normal and obesity groups. 11 DEMs were finally confirmed, including miR-26a-1, miR-210, and so on (Figure 3A). Subsequently, we investigated the relationship between DEMs and OS. We found miR-210 was decreased in obesity group and associated with better OS (Figure 3B). Possible targeted genes of miR-210 were predicted by the TargetScan database (http://www.targetscan.org/vert_72/) and performed with GO and KEGG analysis (Figure 3C, 3D). Interestingly, target genes of miR-210 were enriched in hypoxia associated pathways and annotations (VEGF signaling pathway, HIF-1 signaling pathway, Response to hypoxia). Hypoxia plays a crucial role in cancer metabolism triggering cancer aggressiveness, invasiveness, and treatment resistance. Therefore, we further assessed the hypoxia condition in tumors of normal and obesity groups. Three different hypoxia scoring systems were adopted, including Buffa system [13], Ragnum system [14] and Winter system [15]. We found the hypoxia score of obesity group was less than normal group in all three scoring systems (Figure 3E). Additionally, the expression of miR-210 also showed co-expression with all hypoxia scores derived from different systems (Figure 3F). Finally, we also investigated the Reverse Phase Protein Arrays (RPPA) data and confirmed several reported tumor regulating proteins were differentially expressed in obesity group, including 14-3-3β, GTP-binding protein Di-Ras3 (ARHI), Fatty Acid Synthase (FASN) and ribosomal protein S6 (RPS6, Figure 3G). In summary, downregulated miR-210 in obesity patients repressed hypoxia of tumors, thus prevented the progression of colon cancer.

Subsequently, we researched the role of miR-210 in vitro under both normoxia and hypoxia. Overexpression of miR-210 significantly promoted the invasion and migration ability of HCT-116 under both normoxia and hypoxia. Additionally, overexpression of miR-210 promoted the proliferation ability of HCT-116 only under hypoxia, but not under normoxia. Finally, we investigated hypoxia associated genes (glycolysis associated genes: HK2, HIF1A, PKM, Glut1 and angiogenesis associated genes: VEGFA). Interestingly, overexpression of miR-210 significantly promoted the expression of HK2, HIF1A and VEGFA only under hypoxia. Under normoxia, miR-210 showed no effect at these genes.

### Obesity was associated with the alteration of gene mutations

**Figure 4:**
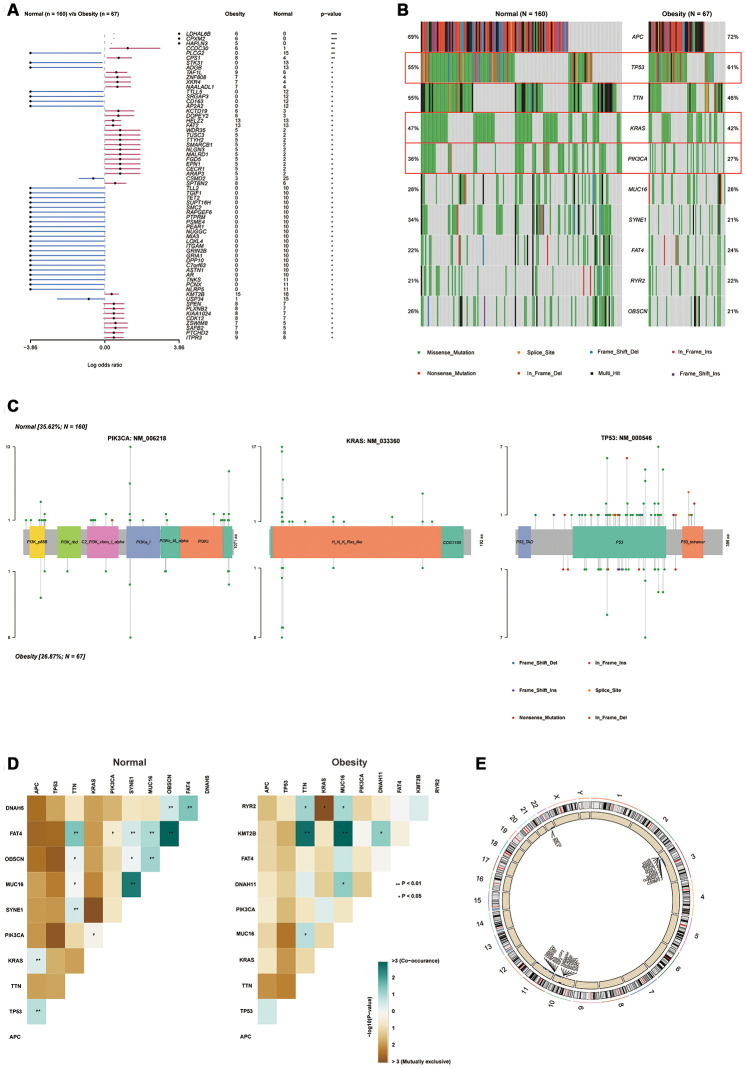
**Obesity was associated with the alteration of gene mutations.** (**A**) Forest plot for different mutations frequency compared between normal and obesity groups, analyzed by Chi-square test. (**B**) Top 10 mutations in normal and obesity groups. (**C**) “Lollipop” graph for specific mutation frequency, types and sites in PIK3CA, KRAS and TP53 (p53) protein domain. Mutation sites were shown on the x-axis, and the frequency of a particular mutation was represented by the height (y-axis). (**D**) Interaction of each top 10 mutation in normal and obesity group analyzed by Fisher exact test. (**E**) The distribution of copy number variation (CNV) in normal and obesity groups analyzed by Chi-square test. (^*^p<0.05, ^**^p<0.01, ^***^p<0.001).

In colon cancer, various oncogene and tumor suppressor gene mutations are reported to be crucial in the development of the disease [16]. Therefore, we further investigated the somatic mutations of colon cancer via R software (“maftools” Package) based on MAF data. We demonstrated an obvious difference between gene mutations frequency in compare of obesity group to normal group. Obesity group showed a higher mutation frequency of LDHAL6B, CPXM2, HAPLN3, and so on. Obesity group also showed a lower mutation frequency of PLCG2, STK31, ADGB, and so on (Figure 4A). Furthermore, we selected 10 most common mutations in colon cancer patients. In the top 10 mutations assessment, the frequency of KRAS and PIK3CA mutations were significantly decreased, whereas the frequency of TP53 mutation were significantly increased in obesity group compared with normal group (Figure 4B). KRAS, TP53 and PIK3CA were important gene mutations in colon cancer that associated with progression and prognosis [17, 18]. We further showed specific mutation sites, types and frequency of them in schematic representations of protein domain information (Figure 4C). Mutation sites were shown on the x-axis, and the frequency of a particular mutation was represented by the height (y-axis). We demonstrated most mutation type of PIK3AC and KRAS was missense mutation in both normal and obesity groups. Obesity group not only have a lower frequency of mutations but also have fewer mutation sites. Compared with PIK3AC and KRAS, TP53 (p53) had various mutation types, including missense mutation, frameshift mutation, in-frame mutation nonsense mutation, and splice-site mutations. Though obesity group showed increased mutation frequency of TP53 compared with normal patients. There was significantly decreased mutation sites in obesity group patients. Especial in TP53 tetramer domain, obesity group showed no mutation site, whereas normal group showed 4 mutation sites, including 2 missense mutations, 1 splice site mutation, and 1 nonsense mutation. Subsequently, we investigated the correlation between each top 10 mutations in two groups (Figure 4D). Interestingly, more co-occurrences between mutations were demonstrated in obesity group compared with normal group (APC and KRAS, PIK3CA and KRAS, etc.) Finally, we investigated the copy number variation (CNV) in two group. Obesity group showed reduced CNV compared with normal group, and the reduction mainly came from chr3, 10, 11, 22 (Figure 4E). In summary, we demonstrated the gene mutation frequency (especial in PIK3CA and KRAS), as well as CNV of obesity groups, were decreased. We inferred the genome of obesity patients was more stable compared with normal patients.

### Obesity was associated with repression of immune checkpoints

**Figure 5:**
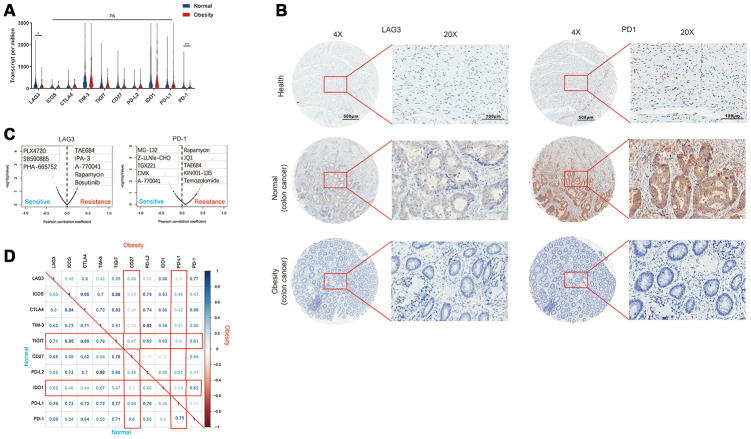
**Obesity was associated with repression of immune checkpoints.** (**A**) The expression of various immune checkpoints in obesity and normal groups. (**B**) The expression of LAG3 and PD-1 in healthy bowel tissues, colon cancer derived from normal (non-obesity) patients and obesity patients was confirmed by immunohistochemistry. (**C**) The association of drug sensitiveness and gene expression (LAG3 and PD-1) analyzed by Genomics of Drug Sensitivity in Cancer (GDSC) database. (**D**) The correlation matrix of each immune checkpoint in obesity and normal group. (^*^p<0.05, ^**^p<0.01).

Immune checkpoints are receptor-based signal cascades that negatively regulate T cells and cause immune tolerance, which allows tumors to evade and escape immune surveillance. The advent of immune checkpoint inhibitors (ICIs) have changed the landscape of cancer treatment. Therefore, we investigated the correlation between immune checkpoints and obesity. Compared with normal group, obesity group showed decreased expression of LAG3 and PD-1 (Figure 5A). Furthermore, the expression of LAG3 and PD-1 in healthy bowel tissues, colon cancer derived from normal (non-obesity) patients and obesity patients was confirmed by IHC (Figure 5B). These results inferred immune surveillance of T cells was activated in obesity patients, thus repressed the growth of tumors. However, decreased expression of immune checkpoints was also associated with the resistance of ICIs. Therefore, we investigated possible replacement through Genomics of Drug Sensitivity in Cancer (GDSC) database (https://www.cancerrxgene.org/). Low expression of LAG3 indicated sensitiveness of PLX4720, SB590885, and PHA-665752. Similarly, low expression of PD-1 indicated sensitivity of MG-132, Z-LLNle-CHO, TGX221, and so on (Figure 5C). Subsequently, we investigated co-expression between each immune checkpoint in obesity and normal groups. In general, all immune checkpoints were significantly co-expressed. It is noteworthy that TIGIT and IDO1 showed stronger co-expression with other immune checkpoints in normal group compared with obesity group (Figure 5D).

Additionally, we also investigated the proportions of different immune cells (22 types) in colon cancer via CIBERSORT algorithm. In all 22 types of immune cells, only gamma delta T cell (γδT) showed significant inhibition in obesity group (Supplementary Figure 1A. 1B). As Interleukins play important roles in immune response, we also investigated the mRNA expression of all Interleukin families. IL11RA and IL17RB were decreased in obesity group, whereas IL19 and IL25 were increased (Supplementary Figure 1C). Among them, low expression of IL11RA also indicated improved OS (Supplementary Figure 1D).

## DISCUSSION

The “obesity paradox” means that obesity increases the risk of cancers, but it may also reduce the mortality and prolong the survival of patients who have suffered cancers. The relevant mechanism behind the paradox remains unclear. Our research aimed to explain the paradox based on multi-omics data derived from TCGA database.

First of all, the existence of obesity paradox in colon cancer was confirmed. Obesity patients (BMI≥30 kg/m^2^) showed improved OS compared with both overweight (25≤BMI<30 kg/m^2^) and normal weight patients (18.5≤BMI<25 kg/m^2^). Subsequently, we combined normal weight and overweight patients as normal group (18.5≤BMI<30 kg/m^2^) for further research. Similarly, obesity group showed improved OS compared with normal group in both Kaplan-Meier analysis and Cox regression model. No significant improvement was determined in DFS and PFS, which may be due to the limited sample size.

Subsequently, we investigated the DEGs between normal group and obesity group. Interestingly, the DEGs were enriched in the digestion and metabolism of various nutrients, including proteins, cholesterol, vitamins, and so on. Previous researches have demonstrated all these mechanisms play important roles in the regulation of tumors. Though most research considered excessive fat, cholesterol, vitamins as risk factors for cancer incidence, whereas rare research focused on cancer progression [19]. In a 20 years follow-up research contains 92,710 individuals, total cancer incidence and total cancer mortality were reported to be negatively correlated to serum cholesterol level [20]. In colon cancer, a low level of cholesterol induces apoptosis of cancer cells without oncogenic Ras mutations [21]. We inferred cancer cachexia reduced the absorption of nutrients, whereas obesity prevented the reduction, and improved nutritional status prolongs the survival of patients. In PPI analysis, SPRR2s, Carboxypeptidase associated genes, KRTs, and Apolipoprotein family were determined to interact (all decreased in obesity group). SPRR2A increases local tumor invasiveness of cholangiocarcinoma [22]. Both CPB1 and CPA1 were reported as susceptible genes of pancreatic cancer [23]. KRT6 promoted the malignant transformation of the immortalized urothelial cells [24]. KRT13 promoted bone and brain metastases of human prostate cancer [25]. High APOA1 and APOB levels and low APOB/APOA1 ratio associated with improved OS [26]. In summary, decreased SPRR2s, Carboxypeptidase associated genes, and KRTs in obesity patients repressed the progression of colon cancer. Furthermore, we also demonstrated the alternation of miRNA in obesity group. miR-210 was significantly decreased in obesity group and associated with improved OS. miR-210 has been reported to be upregulated in hypoxia areas and promote invasion of colon cancer [27, 28]. Similarly, we demonstrated miR-210-targeted genes were enriched in hypoxia associated pathways. Hypoxia induces a metabolic switch from oxidative metabolism to anaerobic glycolysis, thus promotes angiogenesis, neovascularization, and sustained inflammation [29]. Interestingly, we further determined hypoxia was repressed in obesity groups through 3 independent hypoxia scoring systems. Subsequently, we further investigated the role of miR-210 in vitro. We determined overexpression of miR-210 promoted the invasion and proliferation ability of HCT-116 under hypoxia. HIF1A, HK2 and VEGFA were determined to be potential target genes of miR-210. HIF1A and HK2 were important glycolysis regulated genes, VEGFA was tumor angiogenesis gene. They all promoted tumor progression in a hypoxic environment [29, 30]. Therefore, we inferred decreased miR-210 in obesity patients repressed hypoxia related pathways in tumor, thus inhibited the progression of colon cancer.

Oncogenes and tumor suppressors can be either activated or inactivated by mutations such as gene rearrangement, deletion, insertion, or substitution. These mutations affect the prognosis and therapy of colon cancer [31]. Interestingly, we demonstrated the mutations in obesity group showed significant differences compared with normal group. The frequency of KRAS and PIK3CA mutations were significantly decreased in obesity group. Both KRAS and PIK3CA mutations were associated with poor prognosis and chemoresistance of colon cancer patients [32]. Interestingly, the total mutation frequency of TP53 was increased in obesity patients, whereas the specific mutation types and sites were totally different in obesity group. Especially in TP53 tetramer domain, obesity group showed no mutation sites at all, whereas normal group showed 4 mutation sites. TP53 protein functions by forming tetramer and mutations in tetramer domain was reported to its function [33]. Function-lost TP53 protein further repressed normal function TP53 protein. This effect is called dominant-negative effect (DNE). DNE promotes the evolution of precancerous cells and plays an important role in the formation of cancer cells [34]. Therefore, we inferred DNE maybe repressed in obesity patients. Additionally, we also demonstrated obesity patients showed reduced CNV, which indicated the genome of obesity patients may be more stable.

Immune checkpoints play an important role in the field of cancer immunotherapy and are a series of molecules that produce costimulatory or inhibitory signals in the immune response. We demonstrated two important immune checkpoints (PD-1 and LGA3) were downregulated in obesity group. Which indicated immune escape is repressed and the killing effect of T cells is activated in obesity group. Immune cells have been proven to be crucial in the development of various tumors. Different types of tumors have different immune cell subpopulations. Even for the same pathological type, the subpopulation could be different among patients [35]. Therefore, we further investigated the proportions of 22 types of immune cells, and found γδT cells were decreased in obesity group compared with normal group. Previous research has demonstrated γδT cells reduced production of IFN- γ in colon cancer, a main tumor-against factor derived from T cells [36]. Additionally, γδT cells also promote the invasion and proliferation of cancer cells [37, 38]. Previous studies also found that interleukins can regulate tumor microenvironment and participate in tumor development [39–40]. We further investigated the expression of interleukins in colon cancer. We found IL11RA and IL17RB were downregulated, IL19 and IL25 were upregulated in obesity group compared with normal group. Among them, the downregulation of IL11RA was associated with improved OS. Previous research has demonstrated the IL11/IL11RA axis was activated under hypoxia and promoted the proliferation of prostate cancer cells. Combined with miR-210-repressed hypoxia in obesity group, we inferred the inhibition of miR-210-hypoxia-IL11/IL11RA axis in obesity patients repressed the progression of colon cancer.

Unfortunately, there are some deficiencies in our research. Possible biases in the existing "cancer obesity paradox" were reported. In previous research, it was often believed that this paradox only existed in overweight or light obesity, whereas it would be overturned when obesity reached the level of morbid [41]. Although we have adopted 30 kg/m^2^ as the boundary of BMI to avoid bias from overweight, the limited sample size still makes the paradox controversial. However, recent research increased the credibility of our research. In patients with metastatic melanoma treated with targeted drug therapy or ICIs, obesity with BMI above 30 kg/m^2^ almost doubled the PFS and OS compared with BMI within the normal range [42].

In conclusion, we demonstrated obesity prolonged the survival of colon cancer patients. We further revealed relevant mechanisms behind the phenomenon, including hypoxia inhibition, remodeling of gene mutation, alternation of immune checkpoints, and so on.

## MATERIAL AND METHODS

### Data acquisition and processing

All data were derived from the TCGA database. There were 227 cases of colon cancer patients with full Body Mass Index (BMI) data. We downloaded the transcriptome data, copy number data, Reverse Phase Protein Arrays (RPPR) data and mutation annotation format (MAF) from cBioPortal (http://cbioportal.org). Transcriptome data were normalized to gene expression data through the R software package “Limma”. MAF files were summarized, analyzed and visualized through R software package “maftools”. Copy number data and RPPR data can be analyzed directly. Additionally, relevant clinical characteristics were obtained (Table 2).

**Table 2:** Clinical characteristics of patients.

**Factors**		**Number**
Age	≤45	87
	>45	140
Gender	Female	107
	Male	120
Stage	Stage I	32
	Stage II	90
	Stage III	74
	Stage IV	24
T stage	T1	4
	T2	32
	T3	163
	T4	28
M stage	M0	166
	M1	24
	MX	37
N stage	N0	130
	N1	57
	N2	40
BMI (kg/m^2^)	18.5≤BMI<25	77
	25≤BMI<30	76
	BMI≥30	74

MX: indeterminate pathological stage.

### Gene ontology, KEGG pathway, and Protein–protein interaction analysis

Gene list was uploaded to Database for Annotation Visualization and Integrated Discovery (DAVID, david.ncifcrf.gov/) online tool for Gene ontology (GO) and KEGG pathway analysis. Concreate pathways and *P*-value were acquired and visualized by R software. For Protein–protein interaction (PPI) analysis, gene list was uploaded to STRING online tool (https://string-db.org/), genes whose combined score >0.4 were selected and imported into Cytoscape software (Version 3.5.1, The Cytoscape Consoritum, New York, NY, USA) to get visualization.

### Assessment of immune cells

CIBERSORT is a deconvolution algorithm using the expression values of 547 genes to characterize the composition of immune cells in tissues. In this study, we used this algorithm to estimate the relative proportion of 22 infiltrating immune cell types based on gene expression. We uploaded the normalized gene expression data to the CIBERSORT website (http://cibersort.stanford.edu/) and set the algorithm to 1,000 rows. *P*< 0.05 was considered statistically significant [43].

### Patients and specimens

Colon cancer specimens and healthy bowel tissues were collected from July 2017 to July 2019. Patients with the following criteria were excluded from participation: had received adjuvant chemotherapy or radiotherapy prior to surgery; had additional cancers diagnoses. All patients were classified according to the 7^th^ edition of the TNM staging system 23. Postoperative adjuvant therapies were performed, according to standard schedules and doses. All participants gave their written informed consent. This study was approved by the Ethical Committee of Shanghai Pudong Hospital.

### Immunohistochemical (IHC) staining

IHC staining was carried out according to the manufacturer’s instructions. Briefly, formalin-fixed and paraffin-embedded tissue sections were deparaffinized in xylene and hydrated with decreasing concentrations of ethanol (100, 95, 80 and 75%). The slices were then soaked in 10% BSA to inhibit endogenous peroxidase activity and incubated with anti-PD-1 or anti-LAG3 rabbit polyclonal antibody (1:100; Cat: 86163&15372, Cell Signaling Tech, Boston, MA USA) at 4°C overnight. A horseradish peroxidase (HRP)-conjugated rabbit secondary antibody (1:400; Cat: 8114, Cell Signaling Tech) was added for 60 min at room temperature; then, 3,3’-diaminobenzidine development (DAB Substrate Chromogen System; Dako) and hematoxylin staining were performed according to standard protocols. Slides were fixed and images were obtained with an Olympus IX71 inverted microscope and a DP2-BSW Olympus image acquisition software system.

### Cell culture, hypoxia induce and miRNA mimics transfection

Human colon cancer cell lines HCT116 was purchased from the University of Colorado Cancer Center Cell Bank. The cells were cultured in RPMI 1640 medium supplemented with 10% FBS (Invitrogen, Carlsbad, CA, USA) at 37°C in a 5% CO_2_ atmosphere. For hypoxia induce, cells were treated with cobalt chloride (CoCl_2_) which prevents HIF-1α from degeneration by replacing the prolyl hydroxylase (PHD) cofactor Fe^2+^ [44].

The NC mimics and miR-210 mimics were purchased from RiboBio (Shanghai, China). In accordance with the instructions of the product manual, Lipofectamine 3000 (Invitrogen, Inc.) was used to transfect the mimics.

### Cell proliferation assay

3×10^3^ cells suspended in 100ul RPMI-1640 medium were seed into 96-well plate. The cell proliferation was assessed by the CCK8 (Dojindo Molecular Technologies, Japan). 10ul CCK8 solution was given to each well of the plate after different incubation times: 0h, 24h, 48h and 72h. Finally, we measured the absorbance at 450nm wavelength after 2h incubation.

### Cell migration and invasion assays

Cell migration and invasion were analyzed with transwell plates (24-well insert, 8 μm pore size; BD Biosciences, Bedford, MA, USA). The filters (Corning Inc., USA) were coated with (invasion assay) or without (migration assay) 55 μL Matrigel (1:8 dilution; BD Biosciences). The 10^4^ cells were suspended in 100μl RPMI-1640 medium without serum and seeded in the upper chamber. Next, 600μl 90% RPMI-1640 supplement with 10% FBS was added to the bottom chamber. After incubation for 24h, the chambers were fixed by 4% paraformaldehyde for 30 min and then stained by 0.1% crystal violet for 30 minutes. At last, we used a magnification microscope to count the amount of the invasion cells in the bottom of the chamber.

### RNA extraction, reverse transcription and quantitative PCR (RT-qPCR)

Total RNA was extracted by Trizol Regent (Invitrogen) from CRC cells. cDNA was obtained from total RNA with PrimeScript™ RT reagent kit (Takara Bio, Inc., Otsu, Japan). The mRNA expression was assessed by Real-time quantitative PCR, which was carried out in triplicate by a SYBR Premix Ex Taq™ kit (Takara Bio) and ABI 7900HT Real-Time PCR system (Applied Biosystems Life Technologies, Foster City, CA, USA). The primers used are shown in Supplementary Table 1. The comparative cycle threshold values (2-ΔΔCt) were adopted to analyze the final results.

### Statistical analysis

All analyses were performed using SPSS 23.0 and R 3.5.3. All statistical tests were two-sided, and a P value <0.05 was considered statistically significant. Continuous variables that conformed to the normal distribution were compared with the use of an independent t test for comparison between groups, while continuous variables with skewed distribution were compared with the Mann-Whitney U test. Categorical data were tested using the Chi-square test. The correlation matrix was constructed by R software based on Pearson Correlation Coefficient (immune checkpoints) or Fisher exact test (gene mutations). The analysis of survival was performed through the Kaplan-Meier method, which was evaluated by the log-rank test. The univariate Cox regression model was used to analyze the effects of individual variables on survival, and the multivariate Cox regression model was used to discover the independent factors associated with survival.

### Ethics approval

All procedures involving human participants were performed in accordance with Shanghai Pudong Hospital ethical committee and with the 1964 Declaration of Helsinki and its later amendments or comparable ethical standards. All patients provided their written informed consent. The study protocol was approved by the Pudong Hospital Committee on human research.

## Supplementary Material

Supplementary Figure 1

Supplementary Table 1

## References

[1] Long AG, Lundsmith ET, Hamilton KE. Inflammation and colorectal cancer. Curr Colorectal Cancer Rep. 2017; 13:341–51. 10.1007/s11888-017-0373-6 29129972 PMC5678998

[2] Cunningham D, Atkin W, Lenz HJ, Lynch HT, Minsky B, Nordlinger B, Starling N. Colorectal cancer. Lancet. 2010; 375:1030–47. 10.1016/S0140-6736(10)60353-4 20304247

[3] Aleksandrova K, Pischon T, Buijsse B, May AM, Peeters PH, Bueno-de-Mesquita HB, Jenab M, Fedirko V, Dahm CC, Siersema PD, Freisling H, Ferrari P, Overvad K, et al. Adult weight change and risk of colorectal cancer in the European prospective investigation into cancer and nutrition. Eur J Cancer. 2013; 49:3526–36. 10.1016/j.ejca.2013.06.021 23867126

[4] Liu PH, Wu K, Ng K, Zauber AG, Nguyen LH, Song M, He X, Fuchs CS, Ogino S, Willett WC, Chan AT, Giovannucci EL, Cao Y. Association of obesity with risk of early-onset colorectal cancer among women. JAMA Oncol. 2019; 5:37–44. 10.1001/jamaoncol.2018.4280 30326010 PMC6382547

[5] Allott EH, Hursting SD. Obesity and cancer: mechanistic insights from transdisciplinary studies. Endocr Relat Cancer. 2015; 22:R365–86. 10.1530/ERC-15-0400 26373570 PMC4631382

[6] Hakimi AA, Furberg H, Zabor EC, Jacobsen A, Schultz N, Ciriello G, Mikklineni N, Fiegoli B, Kim PH, Voss MH, Shen H, Laird PW, Sander C, et al. An epidemiologic and genomic investigation into the obesity paradox in renal cell carcinoma. J Natl Cancer Inst. 2013; 105:1862–70. 10.1093/jnci/djt310 24285872 PMC3866155

[7] Brunner AM, Sadrzadeh H, Feng Y, Drapkin BJ, Ballen KK, Attar EC, Amrein PC, McAfee SL, Chen YB, Neuberg DS, Fathi AT. Association between baseline body mass index and overall survival among patients over age 60 with acute myeloid leukemia. Am J Hematol. 2013; 88:642–46. 10.1002/ajh.23462 23619915 PMC4214755

[8] Schlesinger S, Siegert S, Koch M, Walter J, Heits N, Hinz S, Jacobs G, Hampe J, Schafmayer C, Nöthlings U. Postdiagnosis body mass index and risk of mortality in colorectal cancer survivors: a prospective study and meta-analysis. Cancer Causes Control. 2014; 25:1407–18. 10.1007/s10552-014-0435-x 25037235

[9] Amptoulach S, Gross G, Kalaitzakis E. Differential impact of obesity and diabetes mellitus on survival after liver resection for colorectal cancer metastases. J Surg Res. 2015; 199:378–85. 10.1016/j.jss.2015.05.059 26115811

[10] Tsang NM, Pai PC, Chuang CC, Chuang WC, Tseng CK, Chang KP, Yen TC, Lin JD, Chang JT. Overweight and obesity predict better overall survival rates in cancer patients with distant metastases. Cancer Med. 2016; 5:665–75. 10.1002/cam4.634 26811258 PMC4831285

[11] Kroenke CH, Neugebauer R, Meyerhardt J, Prado CM, Weltzien E, Kwan ML, Xiao J, Caan BJ. Analysis of body mass index and mortality in patients with colorectal cancer using causal diagrams. JAMA Oncol. 2016; 2:1137–45. 10.1001/jamaoncol.2016.0732 27196302 PMC5016213

[12] Blackburn H, Jacobs D Jr. Commentary: origins and evolution of body mass index (BMI): continuing saga. Int J Epidemiol. 2014; 43:665–69. 10.1093/ije/dyu061 24691955

[13] Buffa FM, Harris AL, West CM, Miller CJ. Large meta-analysis of multiple cancers reveals a common, compact and highly prognostic hypoxia metagene. Br J Cancer. 2010; 102:428–35. 10.1038/sj.bjc.6605450 20087356 PMC2816644

[14] Ragnum HB, Vlatkovic L, Lie AK, Axcrona K, Julin CH, Frikstad KM, Hole KH, Seierstad T, Lyng H. The tumour hypoxia marker pimonidazole reflects a transcriptional programme associated with aggressive prostate cancer. Br J Cancer. 2015; 112:382–90. 10.1038/bjc.2014.604 25461803 PMC4453458

[15] Winter SC, Buffa FM, Silva P, Miller C, Valentine HR, Turley H, Shah KA, Cox GJ, Corbridge RJ, Homer JJ, Musgrove B, Slevin N, Sloan P, et al. Relation of a hypoxia metagene derived from head and neck cancer to prognosis of multiple cancers. Cancer Res. 2007; 67:3441–49. 10.1158/0008-5472.CAN-06-3322 17409455

[16] Pearlman R, Frankel WL, Swanson B, Zhao W, Yilmaz A, Miller K, Bacher J, Bigley C, Nelsen L, Goodfellow PJ, Goldberg RM, Paskett E, Shields PG, et al, and Ohio Colorectal Cancer Prevention Initiative Study Group. Prevalence and spectrum of germline cancer susceptibility gene mutations among patients with early-onset colorectal cancer. JAMA Oncol. 2017; 3:464–71. 10.1001/jamaoncol.2016.5194 27978560 PMC5564179

[17] Arrington AK, Heinrich EL, Lee W, Duldulao M, Patel S, Sanchez J, Garcia-Aguilar J, Kim J. Prognostic and predictive roles of KRAS mutation in colorectal cancer. Int J Mol Sci. 2012; 13:12153–68. 10.3390/ijms131012153 23202889 PMC3497263

[18] Liao X, Morikawa T, Lochhead P, Imamura Y, Kuchiba A, Yamauchi M, Nosho K, Qian ZR, Nishihara R, Meyerhardt JA, Fuchs CS, Ogino S. Prognostic role of PIK3CA mutation in colorectal cancer: cohort study and literature review. Clin Cancer Res. 2012; 18:2257–68. 10.1158/1078-0432.CCR-11-2410 22357840 PMC3628835

[19] Järvinen R, Knekt P, Hakulinen T, Rissanen H, Heliövaara M. Dietary fat, cholesterol and colorectal cancer in a prospective study. Br J Cancer. 2001; 85:357–61. 10.1054/bjoc.2001.1906 11487265 PMC2364063

[20] Törnberg SA, Holm LE, Carstensen JM, Eklund GA. Cancer incidence and cancer mortality in relation to serum cholesterol. J Natl Cancer Inst. 1989; 81:1917–21. 10.1093/jnci/81.24.1917 2593170

[21] Calleros L, Sanchez-Hernandez I, Baquero P, Toro MJ, Chiloeches A. Oncogenic Ras, but not (V600E)B-RAF, protects from cholesterol depletion-induced apoptosis through the PI3K/AKT pathway in colorectal cancer cells. Carcinogenesis. 2009; 30:1670–7. 10.1093/carcin/bgp188 19700418

[22] Specht S, Isse K, Nozaki I, Lunz JG 3rd, Demetris AJ. SPRR2A expression in cholangiocarcinoma increases local tumor invasiveness but prevents metastasis. Clin Exp Metastasis. 2013; 30:877–90. 10.1007/s10585-013-9589-2 23728799

[23] Tamura K, Yu J, Hata T, Suenaga M, Shindo K, Abe T, MacGregor-Das A, Borges M, Wolfgang CL, Weiss MJ, He J, Canto MI, Petersen GM, et al. Mutations in the pancreatic secretory enzymes CPA1 and CPB1 are associated with pancreatic cancer. Proc Natl Acad Sci USA. 2018; 115:4767–72. 10.1073/pnas.1720588115 29669919 PMC5939087

[24] Slusser-Nore A, Garrett SH, Zhou XD, Sens DA, Sens MA, Somji S. The expression of keratin 6 is regulated by the activation of the ERK1/2 pathway in arsenite transformed human urothelial cells. Toxicol Appl Pharmacol. 2017; 331:41–53. 10.1016/j.taap.2017.05.007 28501331 PMC5649386

[25] Li Q, Yin L, Jones LW, Chu GC, Wu JB, Huang JM, Li Q, You S, Kim J, Lu YT, Mrdenovic S, Wang R, Freeman MR, et al. Keratin 13 expression reprograms bone and brain metastases of human prostate cancer cells. Oncotarget. 2016; 7:84645–57. 10.18632/oncotarget.13175 27835867 PMC5356688

[26] Sirniö P, Väyrynen JP, Klintrup K, Mäkelä J, Mäkinen MJ, Karttunen TJ, Tuomisto A. Decreased serum apolipoprotein A1 levels are associated with poor survival and systemic inflammatory response in colorectal cancer. Sci Rep. 2017; 7:5374. 10.1038/s41598-017-05415-9 28710487 PMC5511233

[27] Nijhuis A, Thompson H, Adam J, Parker A, Gammon L, Lewis A, Bundy JG, Soga T, Jalaly A, Propper D, Jeffery R, Suraweera N, McDonald S, et al. Remodelling of microRNAs in colorectal cancer by hypoxia alters metabolism profiles and 5-fluorouracil resistance. Hum Mol Genet. 2017; 26:1552–64. 10.1093/hmg/ddx059 28207045 PMC5393147

[28] Bigagli E, Luceri C, Guasti D, Cinci L. Exosomes secreted from human colon cancer cells influence the adhesion of neighboring metastatic cells: role of microRNA-210. Cancer Biol Ther. 2016; 17:1062–69. 10.1080/15384047.2016.1219815 27611932 PMC5079399

[29] Vaupel P, Mayer A. Hypoxia in cancer: significance and impact on clinical outcome. Cancer Metastasis Rev. 2007; 26:225–39. 10.1007/s10555-007-9055-1 17440684

[30] Carmeliet P. VEGF as a key mediator of angiogenesis in cancer. Oncology. 2005 (Suppl 3); 69:4–10. 10.1159/000088478 16301830

[31] Khambata-Ford S, Garrett CR, Meropol NJ, Basik M, Harbison CT, Wu S, Wong TW, Huang X, Takimoto CH, Godwin AK, Tan BR, Krishnamurthi SS, Burris HA 3rd, et al. Expression of epiregulin and amphiregulin and K-ras mutation status predict disease control in metastatic colorectal cancer patients treated with cetuximab. J Clin Oncol. 2007; 25:3230–37. 10.1200/JCO.2006.10.5437 17664471

[32] Sartore-Bianchi A, Martini M, Molinari F, Veronese S, Nichelatti M, Artale S, Di Nicolantonio F, Saletti P, De Dosso S, Mazzucchelli L, Frattini M, Siena S, Bardelli A. PIK3CA mutations in colorectal cancer are associated with clinical resistance to EGFR-targeted monoclonal antibodies. Cancer Res. 2009; 69:1851–57. 10.1158/0008-5472.CAN-08-2466 19223544

[33] Rollenhagen C, Chène P. Characterization of p53 mutants identified in human tumors with a missense mutation in the tetramerization domain. Int J Cancer. 1998; 78:372–76. 10.1002/(SICI)1097-0215(19981029)78:3<372::AID-IJC19>3.0.CO;2-8 9766574

[34] Boettcher S, Miller PG, Sharma R, McConkey M, Leventhal M, Krivtsov AV, Giacomelli AO, Wong W, Kim J, Chao S, Kurppa KJ, Yang X, Milenkowic K, et al. A dominant-negative effect drives selection of TP53 missense mutations in myeloid Malignancies. Science. 2019; 365:599–604. 10.1126/science.aax3649 31395785 PMC7327437

[35] Becht E, Giraldo NA, Germain C, de Reyniès A, Laurent-Puig P, Zucman-Rossi J, Dieu-Nosjean MC, Sautès-Fridman C, Fridman WH. Immune contexture, immunoscore, and Malignant cell molecular subgroups for prognostic and theranostic classifications of cancers. Adv Immunol. 2016; 130:95–190. 10.1016/bs.ai.2015.12.002 26923001

[36] Meraviglia S, Lo Presti E, Tosolini M, La Mendola C, Orlando V, Todaro M, Catalano V, Stassi G, Cicero G, Vieni S, Fourniè JJ, Dieli F. Distinctive features of tumor-infiltrating γδ T lymphocytes in human colorectal cancer. Oncoimmunology. 2017; 6:e1347742. 10.1080/2162402X.2017.1347742 29123962 PMC5665062

[37] Jin C, Lagoudas GK, Zhao C, Bullman S, Bhutkar A, Hu B, Ameh S, Sandel D, Liang XS, Mazzilli S, Whary MT, Meyerson M, Germain R, et al. Commensal microbiota promote lung cancer development via γδ T cells. Cell. 2019; 176:998–1013.e16. 10.1016/j.cell.2018.12.040 30712876 PMC6691977

[38] Coffelt SB, Kersten K, Doornebal CW, Weiden J, Vrijland K, Hau CS, Verstegen NJ, Ciampricotti M, Hawinkels LJ, Jonkers J, de Visser KE. IL-17-producing γδ T cells and neutrophils conspire to promote breast cancer metastasis. Nature. 2015; 522:345–48. 10.1038/nature14282 25822788 PMC4475637

[39] Zarogoulidis P, Lampaki S, Yarmus L, Kioumis I, Pitsiou G, Katsikogiannis N, Hohenforst-Schmidt W, Li Q, Huang H, Sakkas A, Organtzis J, Sakkas L, Mpoukovinas I, et al. Interleukin-7 and interleukin-15 for cancer. J Cancer. 2014; 5:765–73. 10.7150/jca.10471 25368677 PMC4216801

[40] Voronov E, Carmi Y, Apte RN. The role IL-1 in tumor-mediated angiogenesis. Front Physiol. 2014; 5:114. 10.3389/fphys.2014.00114 24734023 PMC3975103

[41] Lennon H, Sperrin M, Badrick E, Renehan AG. The obesity paradox in cancer: a review. Curr Oncol Rep. 2016; 18:56. 10.1007/s11912-016-0539-4 27475805 PMC4967417

[42] McQuade JL, Daniel CR, Hess KR, Mak C, Wang DY, Rai RR, Park JJ, Haydu LE, Spencer C, Wongchenko M, Lane S, Lee DY, Kaper M, et al. Association of body-mass index and outcomes in patients with metastatic melanoma treated with targeted therapy, immunotherapy, or chemotherapy: a retrospective, multicohort analysis. Lancet Oncol. 2018; 19:310–22. 10.1016/S1470-2045(18)30078-0 29449192 PMC5840029

[43] Chen B, Khodadoust MS, Liu CL, Newman AM, Alizadeh AA. Profiling tumor infiltrating immune cells with CIBERSORT. Methods Mol Biol. 2018; 1711:243–59. 10.1007/978-1-4939-7493-1_12 29344893 PMC5895181

[44] Zhang YB, Wang X, Meister EA, Gong KR, Yan SC, Lu GW, Ji XM, Shao G. The effects of CoCl2 on HIF-1α protein under experimental conditions of autoprogressive hypoxia using mouse models. Int J Mol Sci. 2014; 15:10999–1012. 10.3390/ijms150610999 24945310 PMC4100194

