# Supplementary material for: Colon cancer combined with obesity indicates improved survival- research on relevant mechanism
Source: Aging (Albany NY). 2020 Nov 10;12(23):23778–94. doi: 10.18632/aging.103972 (PMC7762486; doi:10.18632/aging.103972)
Supplement: Supplementary Figure 1 [file aging-12-103972-s001.pdf]

SUPPLEMENTARY TABLE

Supplementary Table 1. The primers of RT-qPCR.

Gene	Forward Primer	Reverse Primer
**Actin**	GGGACCTGACTGACTACCTC	TCATACTCCTGCTTGCTGAT
**PKM**	ACTGGCATCATCTGTACCATTG	AGCCACATTCATTCCAGACTTA
**HK2**	CGACAGCATCATTGTTAAGGAG	GCAGGAAAGACACATCACATTT
**Glut1**	CTGGCATCAACGCTGTCTTC	GCCTATGAGGTGCAGGGTC
**PGK1**	TTCTGTTCTTGAAGGACTGTGT	CTTTAACCTTGTTCCCAGAAGC
**VEGFA**	AGGGCAGAATCATCACGAAGT	AGGGTCTCGATTGGATGGCA
**HIF-1α**	GAACGTCGAAAAGAAAAGTCTCG	CCTTATCAAGATGCGAACTCACA
