# Supplementary material for: Colon cancer combined with obesity indicates improved survival- research on relevant mechanism
Source: Aging (Albany NY). 2020 Nov 10;12(23):23778–94. doi: 10.18632/aging.103972 (PMC7762486; doi:10.18632/aging.103972)
Supplement: Supplementary Table 1 [file aging-12-103972-s002.pdf]

SUPPLEMENTARY FIGURE

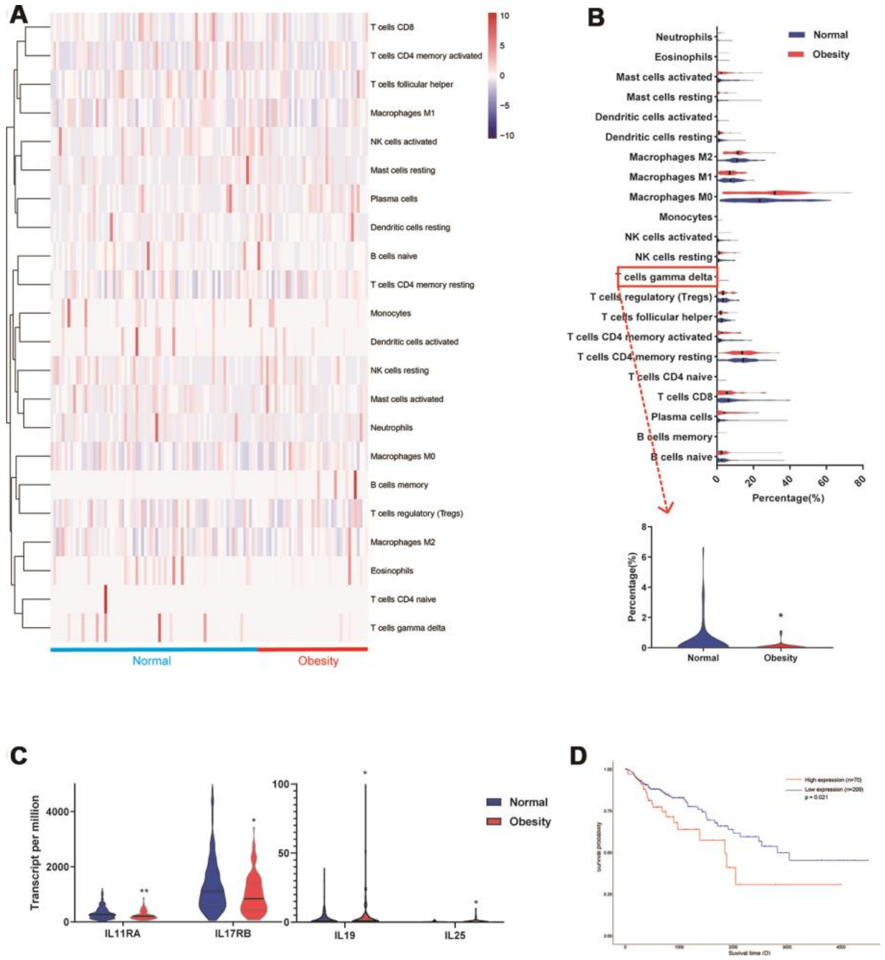

Supplementary Figure 1. Obesity alters tumor infiltrating immune cells and expression of interleukins. (**A**) Heatmap of 22 types immune cells analyzed by CIBERSORT algorithm. (**B**) The proportions of immune cells in normal and obesity groups. (**C**) The mRNA expression of interleukins families in normal and obesity groups. (**D**) The relationship of OS and IL11RA (divided by median of expression) performed by Kaplan-Meier analysis. (*p<0.05, **p<0.01).
